# Can location factors moderate the effects of the demographic composition of SME’s owner managers on the rate of mobile marketing technology (MMT) adoption?

**DOI:** 10.1371/journal.pone.0289734

**Published:** 2023-08-31

**Authors:** Sunday C. Eze, Hart O. Awa, Vera C. Chinedu-Eze, Anthony Eniola

**Affiliations:** 1 Department of Business Administration, Bowen University, Iwo, Nigeria; 2 Department of Marketing, University of Port-Harcourt, Port-Harcourt, Nigeria; 3 Department of Agribusiness and Management, Michael Okpara University of Agriculture, Umudike, Nigeria; 4 Department of Business Administration, Landmark University, Omu Aran, Nigeria; Sri Eshwar College of Engineering, INDIA

## Abstract

Extant scholarly inquiries focused predominantly on the influence of demographic physiognomies of SMEs’ owner-managers on MMT adoption, with dearth recourse to how the proxies of location factors such as workforce diversity and knowledge centres moderate and describe such relationship. To bridge that void, opinions of 345 SMEs’ owner-managers in five industries situated in the state capital and commercial nerve cities of three Geo-political zones of Nigeria were purposively sampled. The analysis involved hierarchical regression and its outcome showed that diversified workforce and enhanced knowledge centres critically moderated the extant relationship between the demographic composition (age, gender, education and experience) of owner-managers and MMT adoption. Hence, location factors are moderators of the relationship between owner/managers’ demographic composition and MMT adoption. Implicit is that understanding the demographic composition of owner-managers provides industry players with strong basis for competitive advantage, as well as opportunity to proactively and tactically make decisions that relate to MMT adoption.

## 1. Introduction

The activity of Mobile Marketing (MM) is on buzz owing to the rapid growth of cloud computing and cloud-based services [1–3], especially given the COVID 19 pandemic [4] that vastly affected the global economy [5]. COVID-19 otherwise known as Corona-virus is an infectious disease that causes pneumonia, severe respiratory problem, kidney failure and ultimately death [4]. Although the global economy has been seriously affected by this deadly disease, contemporary businesses strongly leverage on mobile marketing technology (MMT) to build competitive advantage [6]. The industrial revolution of Industry 4.0 precipitates the development and growth of cloud-based technologies, and explains why myriad of businesses are constantly buying into the potentials of MMT [7]. Globally, the annual surge of MM adoption stood at 4 billion between 2000 and December 2008 [3], and today, more than half of the US consumers use MM [8] while in the UK nearly 72 percent of the consumers use mobile devices [9]. [10] opined that 2/3 of most firms that initiate, develop, adopt and use MMT dedicated over 24 percent of their budget to it. Hence, the vast number of firms that adopt MMT affirm huge market and emerging lifestyle for MM, and ultimately huge scholarship. As the use of MM continues to buzz, small businesses key into its technologies (MMTs) to interact and share contents real-time within and across the firm, especially with the customers and other key trading partners [11,12].

In any economy, large organizations and governments dominant formal development, whereas SMEs informally and more significantly drive the socio-economic development in terms of job creation, industry development, flexibility, innovation and GDP growth [13,14]. SMEs account for over 79 per cent of the economic growth and 96 per cent of all businesses in most countries [15–17]. However, small businesses are nevertheless confronted with the ordeals of adopting cognate MMT, given their operational limitations though the flexibility of MM distinguishes it from other forms of marketing and promotion technologies that offer them (small businesses) the opportunity to compete head-on with large firms [6,18,19]. MM is key to interactive business strategy, SMEs’ diversification and growth, and individual difference factors (IDFs) given the differences in structure, managerial and decision-making styles. Scholars [20,21] recognize that IDFs defined by demographic determinants (e.g., age, experience and functional track, education, and gender) shape MM adoption, because decision-makers’ cognitive perception and assumption about future, alternatives, and consequences reposition corporate destinies. Whereas some studies revealed mixed results [22,23], others [24] argued that geographical location drives digital innovation adoption, even though the dearth of studies that factored the moderation of location between the interactions of the demographic structure of SME owners and adoption of MMT is more suggestive of anecdotal and hunch report.

Supposedly, IT-based innovations bridge the barriers of geographical locations though agglomeration of economies still play critical adoption role, especially in developing economies like Nigeria where uneven developments across zones precipitates classical resources munificence theory and varying adoption opportunities. In the urban cities, for instance, more business activities and opportunities ensue compared to rural areas [25], and MMT’s urge to bridge the obstacles in geographical location through the adoption of MM is hindered by limited focus on upper and middle classes of people [21]. [26] argued that besides the upper and middle classes of people in the cities and/or regions, there are still chunks of low-income earners that are pushing for online businesses especially this period when many businesses are winding up owing to the emergence of Covid-19 pandemic. Covid-19 pandemic rejuvenated business practices for upward trajectory of IT-based innovations and IT-vendors’ boost in market coverage. While extant studies [21,27,28] that correlate demographic determinant of SMEs’ owners and adoption of technology have gained momentum, [21,26] observed that the location of businesses is a cardinal element that shapes adoption behaviour. However, scholars [5,21,24] posit that location factors’ moderation of the interaction between demographic composition of SMEs owners and the rate of MMT adoption seems understudied. [16] and [21] reported that the few studies that investigated the differences in the demographic composition of SMEs owners and the rate of mobile marketing technology adoption were explained by traditional location factors.

Contemporary business practitioners are in dire need of technology applications that boost small business operations even in remote places by improving geographic proximity and overcoming the communications ordeals place by physical distance. Besides, given the agglomeration tendencies in Nigeria and globalization-induced less emphasis on geography and traditional theories of location, it is imperative to further location to enviable levels of measuring how such factors as availability of diverse workforce and knowledge centres moderate the interaction between the demographic composition of SMEs owners and the rate of MMT adoption. Such factors have rarely featured as moderators in many extant conceptual frameworks, especially in home-grown studies and having them as moderators (in the developing economy) lays further credence to the explanatory role of location, generational changes and industrial revolution of Industry 4.0 that deploys cloud-based services to perceive the world as tiny. This suggests that even individuals in highly remote areas now use digital and cloud services to learn, share, connect and build relationship, find work, showcase and promote talent, and build personalized networks. This paper reviewed extant literature, collected analysed data, discussed finding, concluded, and proposed implications for practice and future research.

## 2. Literature review

### 2.1 SMEs and MMT adoption in Nigeria

MM and MMT are of the popular ICT-based innovations that are driving the world in favour of small businesses in terms of customer satisfaction, probably because of their operating agility and innovative spirit. They change the way businesses operate because of their sensitivity and dexterity in using mobile applications to transform business environment to permit ubiquitous transactions any-time and any-way. Mobile marketing involves the use of wireless media as integrated content delivery and direct response vehicle (e.g., cell phones, handheld internet access devices, and digital music players) within a cross-media marketing communications programme [11,29]; while, MMT represents mobile devices or technologies (mobile media, mobile advertising, wireless marketing and wireless advertising) used to communicate and promote offers to customers [30]. They represent the most dynamic, practical and personal marketing strategy; provide continuous and less interrupted access to consumers regardless of time and location [31]; and essential tools that assist businesses to establish real-time interactions with customers and other key stakeholders. However, MMT is swiftly evolving to aid SMEs to key into poverty alleviation and other indices of boosting economies [17–19]; whereas MM is considered an alternative channel to virtual marketing, because it supports small businesses in designing policies that boost promotion strategies and maintain strong customer relationships. These are necessary to aid the global contribution of SMEs in terms of employment generation, development of the economy and the industrial outputs [12,32,33].

Innovations in technology and supports from the government and her agencies have continually caused the informal sector to grow [34,35]. In western countries like Europe, approximately 90% of businesses are small businesses and their contributions in terms of creating jobs, innovation, social, economic and skills development [36,37] cannot be underestimated as they account for nearly 65 per cent of gross added value [38,39]. The growth and expansion of SMEs are obsessively facilitated by the government and its agencies, especially with the surge of investment in ICT and its concomitant paradigm shift in the ways small businesses currently do businesses [40]. ICT-innovations have enhanced the activities of SMEs in Nigeria, especially the service economy because Nigeria’s economy is heavily service-oriented–contributing to the rapid growth and development of the entire economy [14,41]. [42] posited that contemporary efforts to improve Nigeria’s economy is dependent on the service sector. The National Bureau of Statistics (NBS) records that the service sector contributed the largest share of the Gross Domestic Product (GDP) in 2014, with over N8 million which account for over 52 percent; the manufacturing sector contributed over N4 million which represents 27%; and the agriculture sector accounted for over N3 million representing 19 percent of the GDP [43].

MM is important for business growth and access to technology is vital for SMEs to compete with their counterparts in other sectors of the economy [6]. The rise in ICT output has generated new insights into the operations of SMEs. [5] note that 90 percent of SMEs hire MMT to carry out their business activities. However, during MMT adoption, SMEs face both internal obstacles such as business features, costs, and investment returns [44] and external obstacles such as barriers to technology, social, educational, political, legal and regulatory factors [45]. [5] for instance argued that most decisions to adopt mobile devices by SMEs are shaped by owners who solely finance and take decisions alone on which mobile application to invest in without making serious consultations. Others include insufficient market penetration, funding, lack of knowledge of global markets, unfriendly business climes, and inadequate management skills though 90 percent of small SMEs use ICT to execute tasks [35]. Regardless of these obstacles, most businesses in Nigeria use MM as a marketing tool because it is relatively cheap, with low data cost and infrastructure [46]. This suggests why internet usage in Nigeria increased from 62 per cent to 65 per cent, 48 per cent to 53 per cent in Kenya and 48 per cent to 65 per cent in South Africa between 2014 and 2017 [46]. In particular, Nigeria recorded about 187.9 million mobile connections in January 2021, though that increased by 17 million (+10%) between January 2020 and January 2021 accounting for 90.0 percent of the total population. This indicates a continual surge in implementing these devices in Nigeria.

In addition, out of the total population of about 200 million in Nigeria, there are 75 million mobile subscribers with a mobile penetration rate of about 61.53 percent (https://abbakin.com/mobile-marketing-in-nigeria/). Also, the largest mobile market in Africa is Nigeria which accounts for 76% of all internet traffic from mobile applications [41]. Despite the huge growth in this area, the sectors have not witnessed the full economic potential for MMT [47]. The Nigerian government and its agencies (e.g., Central Bank of Nigeria—CBN) have established micro banks, the Small and Medium Enterprise Agency of Nigeria (SMEDAN), and others to assist SMEs to improve their competitiveness. The CBN established a credit guarantee scheme worth N200bn to encourage SMEs and other sectors [35,48]. However, small businesses still face challenges in finding the right technologies to adopt. Even when they adopt, they perceive uncertain on its implementation [13]. The significance of mobile marketing in developing Nigeria economy and the lack of mobile marketing adoption frameworks that may guide service SMEs in adopting and/or implementing mobile marketing devices necessitate this research.

### 2.2 Theoretical underpinning

Many theories explain and predict ICT adoption and behaviour. Among them, **s**cholars [9,18,49–53] noted that the most prominent ones used in the context of small businesses are Theory of Reasoned Action (TRA), Theory of Planned Behaviour (TPB), Resource-based Theory (RBT), Technology Acceptance Model (TAM), Porter’s resource base view models, Technology-Organization-Environment (T-O-E), and Rogers’s Diffusion of Innovation Theory (IDT). Although TRA, TPB, TAM, IDT and Porters models have been used extensively in IT adoption studies, they have been criticised. According to [49] and [54], TRA for instance looks at the behaviour instead of the actions that are immanent to such behaviours. The model further looks at the performance of isolated action or behaviour [55,56] but failed to recognise that there is a propensity to choose among other behaviours. For instance, [9] found out that family members of small businesses, relatives and other external parties coupled with the demographic mechanisms influence SMEs managers’ decision making. TAM focuses on the explanation of attitudes and decisions of small business managers on ICT adoption [57].

Furthermore, [58] argue that the TPB is long mature, and recent studies have constantly extended its core constructs to design and develop other variables to predict behaviour while Porter’s models and T-O-E are mainly apply to large enterprises because their behaviour towards risk taking and economic goals are extraordinary [56]. Such action hampers small businesses in attaining the needed resources [59,60]. RBT rarely recognizes the external actors such as customers, suppliers, and the demographic mechanisms of SMEs characteristics [9]. IDT to a large extent varies from the previous theories because it tends to describe when and how new technologies are accepted, adopted or rejected [53], though some of its constructs overlap with those of TAM. TAM and IDT have been deployed to study both the pre-adoption and post-adoption behaviours and the finding shows that actual and potential adopters of ICT adopted diverse variables that are associated with attitude [61,62]. However, these models rarely undermined the demographic compositions of SME managers. Most importantly, studies [63,64] argued that ICT adoption and development cannot easily be predictable and any single theory that assumes precise predictability would rarely allow researchers to understand the entire processes involved. Thus, researchers often drawn on existing concepts to understand the phenomena to investigate [63], given that a number of concepts have been developed in IT adoption by previous studies [65–67].

Although ample studies [14,15,22,58] investigated the factors that influence MMT adoption in small business context, more inquiries are needed to apply organization and environment dimensions of T-O-E framework to the study of SMEs. Organization and environment dimensions of T-O-E framework are critical on account that extant studies and theories consider management’s physiognomies and supports [22], owners’ eagerness and growth drives [14, and knowledge and characteristics of managers as key adoption predictors of ICT devices. The organization dimension looks at the structures, resources, size of the business and degree of monopolization; and the environment dimension focuses on size of the business, competitors, macroeconomic perspective, and the regulatory background [68–71] According to [72] and [73], these dimensions provide both opportunities and threats for the advancement of technological innovation, and shape the manner micro-businesses implement new technologies. Similarly [74–77] argue that the organization and environment dimensions are very important analytical tools that unravel both the drivers and inherent features of innovation and other environmental circumstances that influence growth and performance. Many studies [14,68,78,79] have been underpinned by these dimensions; thus, providing robust empirical support to the explanation and prediction of technology adoption.

**Fig 1:**
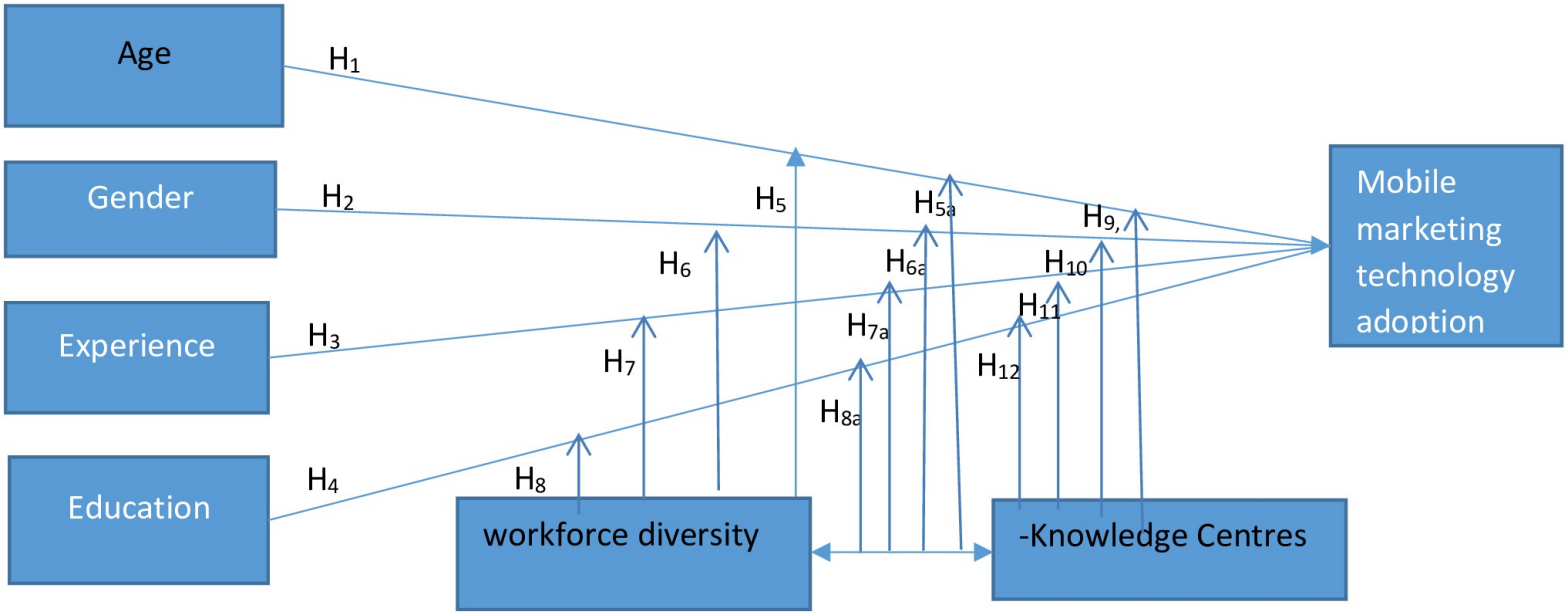
Analytical framework is about here.

This paper adopted organization and environment contexts of T-O-E framework because they were rarely captured by most models, including IDT even though scholars [79,80] posit that the contexts explain intra-firm innovation adoption better. Further scholars [72,80] posit that these dimensions have the capacity to incorporate a large number of variables that are key to a more robust analytical lenses to the explanation and prediction of technology adoption. Often the SME managers and their attitudes towards ICT adoption are influenced by demographic factors such as age, experience, gender, education, and functional tracks. Arguably these variables have been investigated by previous studies in the area of ICT adoption, however, incorporating geographical location as a moderator variable offers a possibly distinctive twist since few studies on ICT adoption. Previous studies [27,28,81,82] investigated these variables in the context of ICT, but the moderating role of geographical location offers a distinct scholarly twist. [12,21] describe location as the vital links between MMT adoption and the demographic characteristics of SMEs managers which may be influenced by both internal and external forces. Scholars [12,15,21,60,62,74] posit that externally, IT experts, vendors and consultants, customers and suppliers, and family members who serve as advisers to SME manager can influence the general attitude, characteristics and decisions of the business. [75] posit that the willingness of SME managers to bring in new applications may be shaped by consumers’ and other trading partners’ attitude and readiness, competitors, regulatory agencies, etc. Hence, this study centred essentially on the demographic characteristics of SME executives/managers such as experience, age, education and gender as depicted in Fig 1 below because these factors were most predominant during the initial evaluation and analysis of the literature.

#### 2.2.1 Age

Age measures a person’s date of birth. [83] and [84] posit there is a variance between the age of decision-makers and the business risk-taking behaviour. Younger managers are more eager to see that businesses entrusted to their care grow [81,85]. The younger the executives, the greater the propensity to take risks because of the emergence of new applications that undertake assignments cost-effectively. Besides younger managers are regularly becoming fluent in their language mobility [86,87]. [88] report that adoption of mobile applications is more pronounced with top managers under 40, where 73 per cent of users within 28 years, 50 years and above use them mainly for communication purposes. Scholars argue that this trend is capable of turning younger managers to device agnostics because of the multi-dimensional ways they use these applications to carry out tasks [21], unlike the older executives that prefer carrying out their task physically and require social enablement for creativity and novelty [85,88,89]. These older managers often consider their career and financial security as the most important to them [5], and often avoid any actions that would disturb their social circles and affect their retirement benefits.

#### 2.2.2 Experience

Experience is a bankable asset to businesses, because when regularly acquired, it impacts on business processes and changes market demands, cost structure, and employees’ productivity and learning curve [5,21]. Several studies [22,90,91] found that lack of internet-use competence and knowledge obstruct adoption. Positive experiences concerning the inconspicuous nature of innovation or perceived ease of use and perceived usefulness shape the use of related technology [92]. When experienced in technology intensifies and strengthens, firms tie together the true prospective of technology application to facilitate business processes and enhance productivity [93]. Studies [93,94] show that managers or top executives’ knowledge has a leaner relationship with how early or late the adoption of new technology applications happens. Therefore, the experience(s) of top executives of SMEs is a major determinant of MMT adoption.

#### 2.2.3 Gender

Scholars opine that the gender of SME managers shapes their risk-taking and strategic thrusts [5], influences individual actions and the decision-making process [95,96], and measures the extent of creativity and/or innovation. [82] and [97] found that innovation adoption originates more from men than from women. Similarly, [82] are of the view that greater variances exist between gender in the adoption of e-mails while [97] found that the association between gender and adoption of ICT is explained by the fact that men use computer facilities more at home than women. Further [88] found that early adopters of new ICT applications are mostly young executives who are predominantly male. Other studies [98] found that in the German mobile technology market, men spend 60 per cent of their time on mobile phones compared to women that spend 40 per cent. This finding may not be the same in most developing nations such as Nigeria where most unemployed women carry different types of mobile devices and spend ample time on phone conversations [21].

#### 2.2.4 Education

The level of educational attainment and professionalism of executives significantly influences ICT adoption [22,99,100]. In proactive and perhaps reactive sense, educational attainment influences ICT decision and affects rapid technology changes. Scholars [101–103] found that educational qualification shapes a person’s value systems to a large extent, intellectual reasoning, learning skill and innovativeness. Highly innovative managers often rely extensively on knowledge and competencies to steer up the firm during uncertainty [18]. Knowledge and well-educated managers are more broad-based and informed about value creation. They often obtain information from scientific sources and experts, show less group cohesiveness, and have the propensity to learn about emerging innovation and to educate others on its benefits. Conversely, executives with weak education are more prone to high levels of risk aversion, because they often feel threatened to novelty.

#### 2.2.5 The Moderators

Conventionally, experts predominantly use geographic factors to design and develop conceptual frameworks and analyse the longitudinal dimension of a business or the complex nature of firms in different geographical areas [5,21]. With the emergence of globalization, less emphasis has been placed on traditional theories of location such as distance (geographical locations) and the barriers of size. One of the importance of technology application is that it assists small businesses located in remote places to improve their geographic proximity and to overcome communications problems hindered by physical distance. Though [104] view that SMEs often adopt ICT applications very slowly, the possibility of their adoption within a region has a significant relationship with the quality of resources located in that area. [105] and [91] found that some demographic locations are more favourable compared to others because they have resource advantages (such as quality educated and varied workforce, good knowledge centres, many venture capital firms and more quality services/service delivery) compared to others.

Furthermore, munificence theory stresses that urban cities arise because of the concentration of people in these cities which are characterised by external forces such as the availability of diverse employees, nature of physical infrastructures and other activities evident in that area. Studies [89,106] propose that availability of diverse employees’ aid the diffusion of new practices for engaging and managing inter-firm relationships, technology information, access to suppliers and other external actors that help firms make up-to-date decisions. The geographic nearness and the institutional framework aid the interaction that will form regional clusters of activities and encourage innovation [98]. This munificence theory and other related theories such as the learning economy approach are of the view that a positive relationship exists between ICT adoption and population density, and such relationship increases as technological complexities of ICTs increase. On the strength of the foregoing discussion, we have the following hypotheses.

H_1_: There is a positive relationship between the executives’ age and MM adoption
H_2_: There is a positive relationship between the executives’ gender and MM adoption
H_3_: There is a positive relationship between the executives’ experience and MM adoption
H_4_: There is a positive relationship between the executives’ education and MM adoption
H_5_: Workforce diversity moderates the relationship between the executives’ age and MM adoption.
H_5a_: Workforce diversity and knowledge centres moderate the relationship between the executives’ age and MM adoption.
H_6_: Workforce diversity moderates the relationship between the executives’ gender and MM adoption.
H6_a_: Workforce diversity and knowledge centres moderate the relationship between the executives’ gender and MM adoption
H_7_: Workforce diversity moderates the relationship between the executives’ experience and MM adoption.
H_7a_: Workforce diversity and knowledge centres moderate the relationship between the executives’ experience and MM adoption.
H_8_: Workforce diversity moderates the relationship between the executives’ education and MM adoption.
H_8a_: Workforce diversity and Knowledge centres moderate the relationship between the executives’ education and MM adoption.
H_9_: Knowledge centre(s) moderates the relationship between the executives’ age and MM adoption.
H_10_: Knowledge centre(s) moderates the relationship between the executives’ gender and MM adoption.
H_11_: Knowledge centre(s) moderates the relationship between the executives’ experience and MM adoption.
H_12_: Knowledge centre(s) moderates the relationship between the executives’ education and MM adoption.

## 3. Research method and strategy

Social science assumes distinct orientations and paradigms on ontology (nature of reality), epistemology (knowing reality), and methods (accessing reality). These prompt Auguste Comte’s positivism and deductivism, and Marx Weber’s vertehen and inductivism as the critical opposing intellectual traditions for the design and collection of data though sometimes, mix approach plays out. Whichever traditions chosen guides the philosophical assumption about the nature and selection of instruments, tools and methods. This paper follows the Auguste’s paradigm because it adopts a realist approach to ontology and supported by positivist epistemology, relative determinism and nomothetic methods. To test the proposed framework, deductive or testing theory approach was adopted; that survey questionnaire was administered to a muilt stage sampling with first a purposive sample of executives/managers of registered small businesses in five industries in the service sector (see Table 1 below) in three geo-political zones of Nigeria—South-East, South-West and South-South. Further, the SMEs sampled must not have more than 20 employees, and owners must directly be part of the management process and capable of exploiting the possibilities of network externalities.

**Table 1:** Cities, number of questionnaire and copies of questionnaire returned.

S/N	Cities	Number of questionnaires administrated	Number of questionnaires returned
1	Port Harcourt	69	36
2	Warri	43	20
3	Enugu	71	42
4	Owerri	43	27
5	Lagos	76	47
6	Ijebu-Ode	43	21
7	**Total**	**345**	**193**

Lagos and Enugu have the highest number of responses because arguably, they are stronger in terms of socio-economic and socio-political strengths, compared to other commercial nerve cities. Cover letter accompanied the questionnaire to guarantee the respondents’ confidentiality on the information provided. The copies of questionnaire returned were 193.

[107] proportional allocation rule was used to determine the proportional copies of instrument from the sample size of 345 executives/managers and [108] table guided the sampling of the respondents. Two cites from each geo-political zone were selected on the grounds of being commercially viable and playing significant host to socio-economic and socio-political activities. Port Harcourt and Warri from South-South; Lagos and Ijebu Ode from South-West; and Enugu and Owerri from South-East. The questionnaire instrument was in the form of structured disguised and structured-undisguised questions, as well as compulsory and non-compulsory questions. The total number of copies of questionnaire distributed, returned and the percentage returns are depicted in Table 1 below.

### 3.1 Measures

Table 2 shows the factor analysis indicating the measurement of instruments of MMT adoption and the moderators’ scales. [21,98] and [21,22,90] developed and purified scales respectively for moderators and MMT. The items were adopted and scaled on Likert-type continuum of 5-point, ranging from "don’t agree at all” to “I completely agree.” The scale items separately describe the constructs of the study for ease of purifying them scientifically. The two location factors that were nucleus of the paper were based on the distinctive nature of Nigeria in terms of development advancements and crude oil deposited in some areas which aid the concentration of business activities. Similarly, numerous employees were perceived by qualification and varied ICT labour, knowledge transfer, experience in terms of managing complex new ICT to maintain inter-firm relations. Knowledge centres measure how organisational frameworks trigger inter-and intra-firm interactions, clusters of inventive ideas and knowledge sharing [21,98].

**Table 2:** Measurement of instruments.

Construct	Items	Meaning Bartlett	KMO	Eigenvalue of the principal Component	% of the variance	Cronbach’s α
diversified workforce	6	p = .000 (significant)	.84	2.65	72.5%	.78
knowledge centres	9	p = .000 (significant)	.75	1.88	62.9%	.68
mobile marketing technology adoption	12	p = .000 (significant)	.78	3.08	72.4%	.81

Age was measured in terms of years (nominal data) and adults are those above 40. Gender is classified in this study as either male or female and represent the nominal scale of 1 if male and 0 if otherwise. Experience measures the consistency in a career with a scale of fewer than 5 years and between 5 and 10 years while education measures academic attainment as shown by [28]: First School Leaving Certificate (FSLC), Junior Secondary Certificate Examinations (JSCE), Senior Secondary Certificate Examinations (SSCE), (WASC) and (GCE), National Diploma (ND) B.Sc., B.Tech, B.A, Higher National Diploma (HND), and Post-graduate degrees. Though studies [21,22,90] confirmed the content validity of most of these items, these items were further exposed to face validity with knowledgeable persons who scrutinized the items to ensure they represent the property measured. A pilot study of 15 executives/managers was carried out to pre-test the scale items on limited scale to improve consistency and comprehension of the properties measured. The collinearity diagnostics were used in the study to check for multi-collinearity. When testing for multicollinearity, the variance inflation factor (VIF) is (1/tolerance). VIF scores should be less than 10, and tolerance scores should be greater than 0.2. All of the values in this analysis have scores greater than 0.2, indicating that there is no threat of multi-collinearity in the study.

### 3.2 Data analysis and presentation

The study used descriptive statistics to examine the overall mean and standard deviation, as well as the distributions of the coefficients of variation (see Table 3). Education and age have the highest mean values among the alternatives under consideration, according to the overall mean values. It shows that a society’s level of education, combined with age, has the greatest influence on MMT adoption, with the highest mean values of 3.14 and 2.52, respectively. Similarly, the standard deviations are greater than one, indicating that there is relatively high variation among respondents regarding the influence and effect of education and age on MMT adoption. Gender, experience, a diverse workforce, and knowledge centres, on the other hand, have distributions with coefficients of variation less than one and are considered low-variance with the distributions.

**Table 3:** Descriptive analysis.

Statistics for Scale	Age	Gender	Experience	Education	diversified workforce	knowledge centres
N	193	193	193	193	193	193
Mean	2.52	1.28	1.78	3.14	1.21	1.58
Standard deviation	1.24	.501	.406	1.09	.416	.642
Minimum	1	1	1	1	1	1
Maximum	7	7	7	7	7	7

### 3.3 Regression analysis

This research work centres on how diversified employees and knowledge centres moderate the relationship between the demographic characteristics of SME top management executives and MMT adoption using hierarchical regression analysis with the following factor-loadings: the independent variables, loading on the direct effects, the moderator, and the interaction effects. Given that some nominal data were gathered, SPSS was deployed to convert them to intervals for the regressions analysis. The summary of the model in Table 4 reveals the predictor variables under the moderating influence of location factors explained an overall estimate of 28 per cent variations of ICT adoption behaviour (see R^2^ = 0.281, p < 0.01). Thus, top management’ physiognomies are mainly a function of demographic and location factors and both shape the adoption of MMT. Furthermore, loading the model variables, the additional variance explained was 1.13 percent and F change of 3.88, p< 0.05), gender (β = 0.215, p < 0.05b), experience (β = 0.236, p < 0.05) and education (β = 0.225, p < 0.05). When the location factors were loaded against age as the moderating factors both separately and jointly, it was revealed that their interactions were significant statistically (see β = 0.414, p < 0.05a; β = 0.323, p < 0.05b; β = 0.280, p < 0.05c). The implication of this is that the link between the age bracket of members of top management of SMEs and MMT adoption is influenced by the location factors (β = 0.274, p < 0.05) and workforce less than 40 are main adopters. In addition, when location factors moderated the relationship between experience and MMT, separately and jointly, their interactions were statistically significant (see β = 0.225, p < 0.05a; β = 0.218, p < 0.05b; β = 0.226, p < 0.05c). This result indicates that top management executives with at least 5 years of experience were very crucial in the adoption decision of MMT. When the location factors moderated the association between gender and MMT, their interactions both separately and jointly were statistically significant (β = 0.216, p < 0.05a; β = 0.313, p < 0.05b; β = 0.234, p < 0.05c). Furthermore, location factors moderated the relationship between education and MMT separately and jointly. The results show a positive moderation and positive statistical interactions at (β = 0.225, p < 0.05a; β = 0.312, p < 0.05b; β = 0.201, p < 0.05c). This implies that that B.Sc./HND and above (β = 0.207, p < 0.05) have a significant influenced on MMT adoption. There was an incremental variance of 15 per cent brought by the location factors and 32 per cent incremental variance when the interaction items were loaded. The F change statistics of 3.07 for model 3 and 4.07 for model 4 were significant.

**Table 4:** Hierarchical regression analysis.

Independent Variable	Std. Beta stage1	I Std. Beta stage2	Std. Beta stage3	Std. Beta stage4
Age	0.255*	0.212**	0.281**	0.224*
Gender	0.215*	-0.193	-0.156*	0.242*
Experience	0.236*	0.423	0.201*	0.253*
Education	0.225*	0.453	0.123	0.214*
**Direct Effects**:				
** H**_**1**_: **Age and MMT**				
** **✓ Adoption is at less than 40 years	0.274*			
** **✓ Adoption is faster at 40 years	0.423			
** **✓ Adoption is faster at above 40 years	0.231			
** H**_**2**_: **Gender and MMT**				
** **✓ Adoption is faster when males are involved		0.263*		
** **✓ Adoption is faster when females are involved		0.285		
** H**_**3**_: Experience **and MMT**				
** **✓ Adoption is faster when experience is at least 5 years			0.265*	
** **✓ Adoption is faster when experience is less than 5 years			0.244	
** **✓ Adoption is faster when experience is between 5 and 10 years.			0.431	
** H**_**3**_: Education **and MMT**				
** **✓ Adoption is faster when education is at least OND.				0.234
** **✓ Adoption is faster when education is B.Sc./HND and above.				0.207*
**Moderating Variables**				
** **✓ **H**_**5**_: Age, diversified workforce, MMT				0.414*
** **✓ **H**_**6**_: Gender, diversified workforce, MMT				0.216*
** **✓ **H**_**7**_: Experience, diversified workforce, MMT				0.225*
** **✓ **H**_**8**_: Education, diversified workforce, MMT				0.210*
** **✓ **H**_**9**_: Age, knowledge centres, MMT				0.323*
** **✓ **H**_**10**_: Gender, knowledge centres, MMT				0.313*
** **✓ **H**_**11**_: Experience, knowledge centres, MMT				0.218*
** **✓ **H**_**12**_: Education, knowledge centres, MMT				0.312*
**Interaction of the Two Variables**				
** **✓ **H**_**5a**_: Age, diversified workforce, knowledge centres and MMT			0.021	0.280*
** **✓ **H**_**6a**_: Gender, diversified workforce, knowledge centres and MMT			0.065	0.234*
** **✓ **H**_**7a**_: Experience, diversified workforce, knowledge centres and MMT			0.237	0.226*
** **✓ **H**_**8a**_: Education, diversified workforce, knowledge centres and MMT			0.165	0.201*
**Model Summary**				
R	0.521	0.554		0.602
R^2^	0.281	0.310		0.362
Adjusted R^2^	0.227	0.272	0.256	0.226
R^2^ Change	-	0.045		
F Change	-	3.52**	3.307**	0.407**

Note

*p<0.05;

**p<0.01.

## 4.0 Discussion

This study investigated how knowledge centres and diverse workforce moderate the relationship between the demographic structure of top executives SMEs and MMT adoption. The relationships between the demographic constructs and MMT adoption were also discussed as well as how such relationships were influenced by the moderator variables.

The results showed that age has a significant positive relationship with MMT adoption. While this is consistent with other works [5,15,109], the study revealed that younger executives play significant roles in the adoption of MMT. However, this finding contradicts others studies [22] that established that age has a significant negative effect on technology adoption. The reason for this contradiction may be as a result of the fact that technology may be adopted across ages in the western countries compared to developing economies where adoption is faster with younger managers and executives who often take advantage of it to compete effectively. When location factors (diversified workforce and knowledge centres) were loaded against age, their interactions were statistically significant (see β = 0.414, p < 0.05a; β = 0.323, p < 0.05b). This finding implies that the relationship between the age bracket of members of top executives of SMEs and MMT adoption is influenced by location factors (see β = 0.274, p < 0.05). Executives who are less than 40 are key adopters and are often seen more in cities than in rural areas. Although evidence suggests that executives who are less than 40 are the key adopters [26], several studies [22,81] are of the view that age is linked to the strategic and proactive nature of top management in dealing with competition and proximity.

Furthermore, the finding revealed that the experiences of the top executive of SMEs play a major role in determining MMT adoption. This finding is in line with previous works [5,15,110] that found that top management’s, skills, internet-use skills and practices trigger the adoption of technology applications. Employee’s gratitude for the potentials of MMT platforms is directly linked to executive learning and experience curves, which certainly reduces the difficulties of adopting and implementing new MMT. The study also revealed that when location factors was measured against experience, their interactions were statistically significant (see β = 0.225, p < 0.05a; β = 0.218, p < 0.05b). This implies that top management executives with at least 5 years of experience are core in the adoption and implementation of MMT. Also, several previous works [16,17,23,81] believe that managerial experience explains the tendency to comprehend and adopt technology applications that aid competition.

The finding also shows that gender has a significant positive correlation coefficient with MMT adoption and the value for males was statistically significant (see (β = 0.216, p < 0.05a; β = 0.313, p < 0.05b). It was found that males use such applications to aid productivity while female mostly uses it as application packages which are in line with [102] that found that genders differences influence the use technology applications. This is consistent with the works of [82,97] and [27] that found statistical interaction between e-commerce adoption and gender. In addition, when diversified workforce and knowledge centres were factored in, their interactions show that they are statistically significant.

Finally, there was a significant positive correlation coefficient with ICT adoption and education which is consistent with [21] and [101] and [91] studies. For instance, [101] investigated broadband application use in families and found that such applications are shaped by the executive knowledge of the benefits of the application and the expectation of the business instead of the educational attainment of the decision-makers. Although the finding was inconsistent with previous studies with [92] however, when diversified workforce and knowledge centres were measured against education, there were positive statistical interactions.

## 5. Conclusion and implication

The location of a business is different concerning the opportunities it offers to diverse young, sophisticated, and experienced employees, which eventually describe the degree of MMT adoption of a firm. The findings show that diversified workforce and knowledge centres directly shapes top executives demographic structure and MMT adoption. While small businesses that are situated in rural or semi-urban cities adopt MMT solutions very slowly compared to those in major cities due to limited available resources that occasionally encourage adequate MMT adoption, [21] are of the view that small businesses require quality resources to expand geographical proximity and improve communications challenges because of their size and physical distance.

Having recognised that corporate directions are mainly a product of cognitive insights and expectations of major players, this study investigated how the relationship between the demographic structure of top management executives and MMT adoption is moderated by location factors (diversified workforce and knowledge centres). In conclusion, findings from this research suggest that the availability of diversified employee and knowledge centres statistically moderate the age, gender, education, and experience structure of top executives and MMT adoption. Although MMT has not attained its target in some areas because of the uneven distribution of infrastructural facilities. That is the main reason some places play robust hosts than others thus this study has some implications.

First, the government should develop and build facilities and infrastructures that are not only evenly distributed but also spread such facilitates and infrastructures to discourage rural-urban migration and inspire small businesses to take advantage of the full potentials of MMT regardless of where they are situated especially during this period the entire world is recovering from the deadly Covid-19 disease. Second, small businesses should understand and recognize the variances in the adoption and demographic structure of top executives as suggested by location factors and make decisions that will meet their objectives. Third, small businesses should engage their staff in different learning exercises which should be carried out regularly. This could be achieved by creating an atmosphere that permits training and retraining in a bid to unveil other opportunities beyond the present location.

### 5.1 Limitations and further research

The sample of this study was drawn from three geo-political zone in Nigeria (South-east, South-west, and South-south) using survey method, the generalisation of the finding across the six geo-political zones in Nigeria was not fully ascertain. Hence, further studies should adopt mix method approach to investigate all the six geo-political zones in Nigeria zones since no two or three zones are precisely the same. In addition, approaches adopted in this research are subjective in nature. This creates room for errors leading to bias, however, a number of steps were presented to ease the effect on the results.

Secondly, the study only investigated experience, age, education and gender in relation to the moderating effects, further studies should consider marital status, income and other variables.

Finally, future research could explore the impact of other location factors, such as local competition, cultural factors, and regulatory frameworks, on MMT adoption, investigate the role of other firm-level factors, such as organizational culture and innovation capacity, in shaping MMT adoption and examine the potential synergies and trade-offs between MMT adoption and other sustainable development goals, such as reducing carbon emissions and promoting social inclusivity.
